# Stroma-Mediated Resistance to S63845 and Venetoclax through MCL-1 and BCL-2 Expression Changes Induced by miR-193b-3p and miR-21-5p Dysregulation in Multiple Myeloma

**DOI:** 10.3390/cells10030559

**Published:** 2021-03-04

**Authors:** Esperanza M. Algarín, Dalia Quwaider, Francisco J. Campos-Laborie, Andrea Díaz-Tejedor, Pedro Mogollón, Elena Vuelta, Montserrat Martín-Sánchez, Laura San-Segundo, Lorena González-Méndez, Norma C. Gutiérrez, Ramón García-Sanz, Teresa Paíno, Javier De Las Rivas, Enrique M. Ocio, Mercedes Garayoa

**Affiliations:** 1 Cancer Research Center (IBMCC-CSIC-USAL), University Hospital of Salamanca (IBSAL), 37007 Salamanca, Spain; macalgpac@usal.es (E.M.A.); dalia@usal.es (D.Q.); adiaz062@usal.es (A.D.-T.); pmog@usal.es (P.M.); elena.vuelta.r@usal.es (E.V.); monseratt@usal.es (M.M.-S.); duckey@usal.es (L.S.-S.); lgonzalez@usal.es (L.G.-M.); normagu@usal.es (N.C.G.); rgarcias@usal.es (R.G.-S.); tpaino@usal.es (T.P.); 2 Bioinformatics and Functional Genomics Group, Cancer Research Center (CIC-IBMCC, CSIC/USAL/IBSAL), Consejo Superior de Investigaciones Científicas (CSIC), University of Salamanca (USAL) and Institute for Biomedical Research of Salamanca (IBSAL), 37007 Salamanca, Spain; fjc38@cam.ac.uk (F.J.C.-L.); jrivas@usal.es (J.D.L.R.); 3 The Gurdon Institute (Wellcome Trust/Cancer Research UK), University of Cambridge, Cambridge CB2 1QN, UK; 4 Center for Biomedical Research in Network of Cancer (CIBERONC), 28029 Madrid, Spain; 5 University Hospital Marqués de Valdecilla (IDIVAL), University of Cantabria, 39011 Santander, Spain; ocioem@unican.es

**Keywords:** multiple myeloma, BH3-mimetics, mesenchymal stromal cells, miR-193, miR-21, anti-apoptotic proteins

## Abstract

BH3-mimetics targeting anti-apoptotic proteins such as MCL-1 (S63845) or BCL-2 (venetoclax) are currently being evaluated as effective therapies for the treatment of multiple myeloma (MM). Interleukin 6, produced by mesenchymal stromal cells (MSCs), has been shown to modify the expression of anti-apoptotic proteins and their interaction with the pro-apoptotic BIM protein in MM cells. In this study, we assess the efficacy of S63845 and venetoclax in MM cells in direct co-culture with MSCs derived from MM patients (pMSCs) to identify additional mechanisms involved in the stroma-induced resistance to these agents. MicroRNAs miR-193b-3p and miR-21-5p emerged among the top deregulated miRNAs in myeloma cells when directly co-cultured with pMSCs, and we show their contribution to changes in MCL-1 and BCL-2 protein expression and in the activity of S63845 and venetoclax. Additionally, direct contact with pMSCs under S63845 and/or venetoclax treatment modifies myeloma cell dependence on different BCL-2 family anti-apoptotic proteins in relation to BIM, making myeloma cells more dependent on the non-targeted anti-apoptotic protein or BCL-X_L_. Finally, we show a potent effect of the combination of S63845 and venetoclax even in the presence of pMSCs, which supports this combinatorial approach for the treatment of MM.

## 1. Introduction

Multiple myeloma (MM) is an incurable hematological disorder characterized by the accumulation of malignant plasma cells in the bone marrow (BM). Although the introduction of targeted therapies in recent years has significantly prolonged the survival of MM patients [1,2], most of them ultimately relapse or develop resistance to therapy. It is well known that the tumor-associated BM microenvironment (e.g., stromal cells, immune components, osteoblasts, osteoclasts, endothelial cells.) plays a key role in MM, promoting disease progression and drug resistance [3,4]. Therefore, the development of novel agents that are not only based on the biology of the tumor cell but also effective within the BM microenvironment is of the utmost importance [5,6].

Among the non-tumor components residing in the BM, mesenchymal stromal cells (MSCs) establish important dynamic interactions with MM cells either through cell to cell adhesion, by soluble factors, or via extracellular vesicles [3,7]. This results in the deregulation of several signaling pathways in MM cells, ultimately leading to tumor cell growth, migration, survival, and drug resistance [3,4]. In fact, after interaction with BM-MSCs, multiple transcripts belonging to a great diversity of molecular networks (oncogenic kinases, cytokines, chemokines and chemokine receptors, oncogenic transcription factors, cell cycle regulators, and anti-apoptotic family members, among others) are differentially expressed in MM cells [8]. At post-transcriptional level, BM-MSCs partly exert their regulatory effects on MM cells through miRNAs which affect the expression of their target mRNAs [9]. For instance, Hao et al. reported that interleukin 6 (IL6) secreted by BM-MSCs led to miR-15a/-16 suppression in MM cells, protecting them from apoptosis induced by bortezomib [10]. Similarly, adherence to BM-MSCs has been found to increase miR-21 expression in MM cells, conferring resistance to apoptosis triggered by dexamethasone, doxorubicin, and bortezomib [11]. Levels of miR-125b, which targets IRF4 mRNA, were downregulated in MM cells after stromal interaction, and this promoted myeloma growth and survival [12]. Gulla et al. observed overexpression of miR-221/222 in MM cells when co-cultured with BM-MSCs, and miR-221/222 inhibition overcame melphalan resistance of tumor cells [13]. 

Despite progress achieved in therapeutic options in the last two decades, MM remains an incurable disease and efforts continue to find new molecules to be added to the anti-MM armamentarium [14]. Within these, BH3-mimetics with structures based on the BH3 domain from certain pro-apoptotic proteins are emerging as promising drugs for hematologic malignancies. BH3-mimetics bind with high affinity to specific anti-apoptotic proteins (e.g., MCL-1, BCL-2, or BCL-X_L_), promoting the release of pro-apoptotic BCL-2 family members which ultimately engage BAX/BAK effectors to induce apoptosis [14,15]. The first BH3-mimetic with high anti-tumor activity was the dual BCL-2/BCL-X_L_ inhibitor ABT-737 [16], whose diminished efficacy observed in MM cells when co-cultured with BM-MSCs was partially reverted by IL6 blockage [17]. An orally bioavailable analog, ABT-263 (navitoclax), was subsequently developed; however, the thrombocytopenia associated with BCL-X_L_ inhibition precluded the further clinical development of this agent [18]. For this reason, a selective BCL-2 inhibitor ABT-199, also known as venetoclax, was generated next and proven to be effective predominantly in the subgroup of MM patients harboring the t(11;14) translocation [19]. However, since MM cells generally overexpress MCL-1 [20] and require MCL-1 for survival [21,22], several BH3-mimetics targeting this anti-apoptotic protein have been recently developed [23,24,25] and are currently being tested in preclinical and early phase clinical trials. Among them, S63845 has shown higher affinity than previous MCL-1 inhibitors, displaying a strong anti-MM effect in preclinical studies as a single agent and in combination with venetoclax [23,26]. 

It has been reported that the BM microenvironment, and specifically IL6 derived from BM-MSCs, shifts dependency towards MCL-1 in MM cells [17]. This is majorly attained through phosphorylation of the pro-apoptotic protein BIM, which switches its binding from BCL-2 or BCL-X_L_ to MCL-1, and in some cells through upregulation of MCL-1 expression by STAT-3 [17,27,28]. However, the possibility of post-transcriptional regulation of anti-apoptotic proteins through miRNAs in the context of the BM microenvironment, and the influence that this may have on the activity of BH3-mimetics, has been barely studied. In this work, we show that the stromal BM microenvironment modifies the efficacy and the mechanism of action of S63845 and venetoclax, partly as a consequence of the altered expression of miR-193b-3p and miR-21-5p observed in MM cells after the interaction with pMSCs (patient-derived MSCs). Remarkably, the combination of S63845 with venetoclax is able to overcome the proliferative advantage conferred by pMSCs.

## 2. Materials and Methods

Drugs: S63845 was purchased from MedChemExpress (Monmouth Junction, NJ, USA) and venetoclax from LC Laboratories (Woburn, MA, USA)

Cell lines, primary samples, and cultures: The human cell lines MM.1S and NCI-H929 were purchased from ATCC (Manassas, VA, USA), whereas RPMI8226, JJN3, KMS12-BM, and HEK293 were obtained from DSMZ (Braunschweig, Germany). The origin of the MM1S-luc cell line (stably expressing luciferase) has been explained previously [29]. Authentication and in vitro growth conditions of MM cell lines have already been described [29]. BM samples from MM patients were obtained following approval from the University Hospital of Salamanca Review Board and after written informed consent from patients. Research with human samples was conducted in accordance with ethical standards and principles expressed in the Declaration of Helsinki. pMSCs from bone marrow were isolated and expanded as previously reported [29] and used to establish co-cultures of MM cell lines and pMSCs. Specifically, MM.1S-luc and pMSC co-cultures in 96-well plates were used to test drug cytotoxicity [6,29]. For Western blot and qRT-PCR analyses, 2 × 10^5^ primary BM-MSCs were plated overnight in 60 cm^2^ plates and 6 × 10^6^ MM cells were then added and co-cultured for 48 h. MM cells were recovered by carefully flushing for protein extraction or RNA expression analyses. 

Immunoblotting: Cells were collected, washed with PBS, and lysed in ice-cold lysis buffer (140 mM NaCl, 50 mM EDTA, 10% glycerol, 1% NP-40, 20 mM Tris HCl, pH 7) with protease (sc-29130) and phosphatase (sc-45045) inhibitors from Santa Cruz Biotechnology (Dallas, TX, USA). Protein extracts were boiled in 4X electrophoresis sample buffer and resolved in 10% SDS-PAGE gels. After electrophoresis, proteins were transferred to Immobilon transfer membranes (Merck Millipore, Burlington, MA, USA), which were subsequently blocked for 1 h with 1% BSA TBST solution (Tris-buffered saline, 0.1% Tween 20). Membranes were incubated overnight with a primary antibody. After washing three times with TBST, membranes were incubated with a horseradish peroxidase (HRP)-conjugated appropriate secondary antibody. Bands were visualized by a luminol-based detection system using Clarity Western Peroxide and Luminol/Enhancer Reagents (Bio-Rad, Hercules, CA, USA). All antibodies were purchased from Cell Signaling Technology (MCL-1 ref 5453, BCL-2 ref 4223, BCL-X_L_ ref 2762, BIM ref 2933, HRP-conjugated secondary antibodies refs 7074 and 7076; Danvers, MA, USA), except for anti-α-tubulin (Calbiochem ref CP06). 

Immunoprecipitations: MM cells were harvested in lysis buffer with protease and phosphatase inhibitors (Santa Cruz Biotechnology). Protein levels were quantified by Bradford assay, and equal concentrations of cleared lysates were subjected to immunoprecipitation with an anti-BIM antibody (Cell Signaling Technology ref 5453). Immunocomplexes were captured through incubation with protein-A sepharose beads (Sigma-Aldrich, St. Louis, MO, USA) overnight at 4 °C. Beads were washed, boiled in SDS sample buffer, and the immunoprecipitates were analyzed by immunoblotting.

Cell cytotoxicity: MM.1S-luc cells alone or in co-culture with pMSCs were treated with S63845, venetoclax, or their combination for 48 h. After the addition of luciferase, bioluminescence of MM.1S-luc cells was considered a surrogate of cell viability. Half-maximal inhibitory concentration (IC_50_) for each drug was calculated using SigmaPlot graphing 14.5 software. Alternatively, apoptosis after treatment with S63845, venetoclax, or their combination was measured by flow cytometry in RPMI8226, JJN3, and KMS12-BM cells in monoculture or after co-culture with pMSCs, using an Annexin V/PI assay kit (Immunostep, Salamanca, Spain).

Quantitative real-time PCR (qRT-PCR): Total RNA was isolated using the Direct-zol and RNA Miniprep kit (Zymo Research, Irvine, CA, USA), according to the manufacturer’s instructions. Purity and concentration of isolated RNA were determined by Agilent 2100 Bioanalyzer (Agilent Technologies, Santa Clara, CA, USA). miRNA expression was determined by qRT-PCR (TaqMan Advanced miRNA Assays for hsa-miR-193b-3p (478314_mir), hsa-miR-21-5p (477975_mir), and hsa-miR-423-5p (478090_mir; used for normalization) (Applied Biosystems, Foster City, CA, USA)) following the manufacturer’s protocol. Data were calculated using the 2^−ΔCt^ method. 

Transfections: Cell lines were transfected using the Nucleofector II System (Lonza, Basel, Switzerland) with G-16 (MM.1S) and A-23 (HEK293) programs. Cells were transfected with miRIDIAN microRNA Mimics or Negative Non-Targeting Control#1 (Dharmacon, Lafayette, CO, USA); miRCURY LNA Power Inhibitors or Negative Control A (Exiqon); and pmirGLO dual-luciferase reporter vector (Promega, Madison, WI, USA). Cells were harvested 48 h after transfection for protein extraction or to test the efficacy of S63845 and venetoclax.

Luciferase reporter assay: Double-stranded DNA oligonucleotides containing the wild-type (WT) or mutant (MUT) miR-193b-3p and miR-21-5p binding sites in the 3′-UTR of MCL1 and BCL2 mRNAs were ligated at PmeI and Xbal restriction sites of the pmirGLO dual-luciferase reporter vector (Promega). For luciferase assays, HEK293 cells were co-transfected with 500 ng of plasmid constructs and 50 nM of corresponding miRNA or negative control (NC) mimics. Cells were collected 24 h after transfection, and firefly and renilla luciferase activities were measured using the Dual-Luciferase Reporter Assay (Promega) following the manufacturer’s protocol.

## 3. Results

### 3.1. Co-Culture of MM.1S Cells with pMSCs Alters the Cytotoxic Effect of S63845 and Venetoclax in Monotherapy

**Figure 1:**
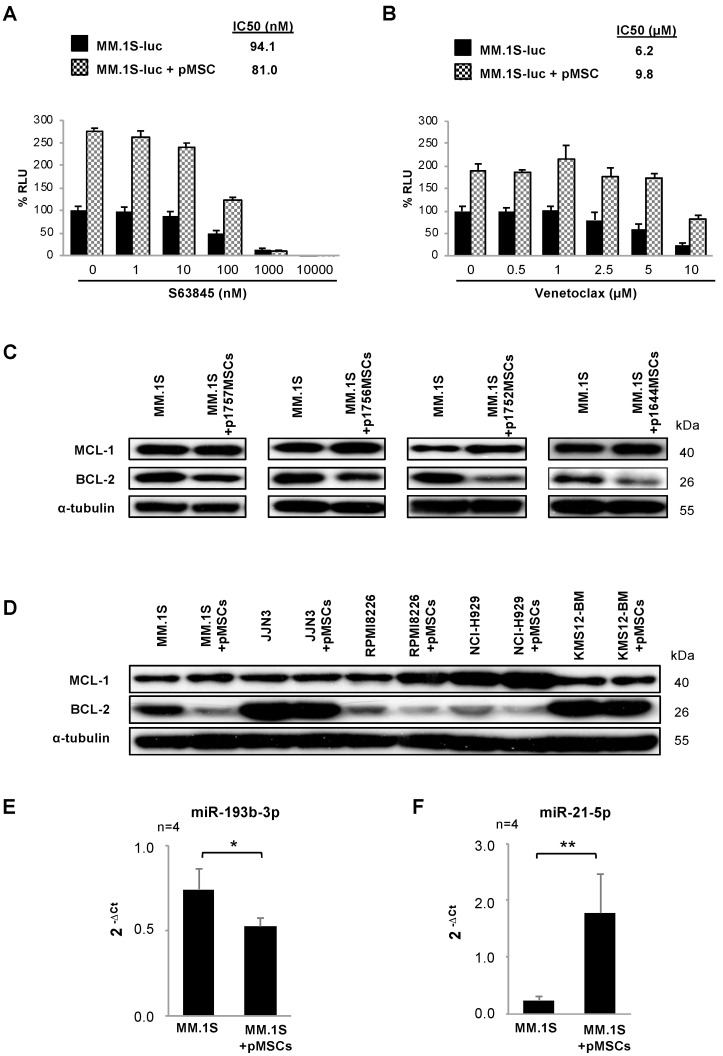
Patient-derived mesenchymal stromal cells (pMSCs) modify the efficacy of S63845 and venetoclax, MCL-1 and BCL-2 levels, and the expression of miRNAs potentially regulating these proteins in multiple myeloma (MM) cells. (**A**,**B**) MM.1S-luc cells were co-cultured with pMSCs for 48 h with S63845 (**A**) or venetoclax (**B**) at the indicated doses. MM.1S-luc growth was assessed by luciferase bioluminescence signal, which was normalized relative to the growth of MM.1S-luc cells alone and in the absence of drug treatment. Graphs show the mean (*n* = 3) ± SD. (**C**) Immunoblotting analysis of MCL-1 and BCL-2 in MM.1S cells in monoculture and co-culture with pMSCs from four MM patients. α-tubulin was used as a loading control. (**D**) Western blot evaluation of MCL-1 and BCL-2 in MM.1S, JJN3, RPMI8226, NCI-H929, and KMS12-BM cells cultured in the absence and presence of pMSCs (from the same patient). α-tubulin was used as a loading control. (**E**,**F**) Normalized expression of miR-193b-3p (**E**) and miR-21-5p (**F**) in MM.1S cells alone or co-cultured with pMSCs as assessed by qRT-PCR. Results are expressed as the mean ± SEM. Student’s *t*-test (*, *p* < 0.05; **, *p* < 0.01).

Considering the critical role of the stromal BM microenvironment in mediating drug resistance, first we wanted to investigate whether the co-culture of MM cells with pMSCs modified the anti-myeloma effect of S63845 or venetoclax. For this purpose, co-cultures of MM.1S-luc cells and pMSCs were exposed to increasing concentrations of S63845 (1–10,000 nM) or venetoclax (0.5–10.0 µM) for 48 h. Despite the proliferative and presumed protective advantage conferred by pMSCs, S63845 (Figure 1A) and venetoclax (Figure 1B) were able to reduce MM cell viability in a dose-dependent manner. However, the presence of tumor-associated pMSCs reduced the IC_50_ value of S63845 in MM.1S-luc cells from 94.1 to 81.0 nM, whereas it raised that of venetoclax from 6.2 to 9.8 µM. Importantly, neither S63845 nor venetoclax affected pMSC viability, even at high concentrations (Figure S1A,B).

### 3.2. pMSCs Modify the Expression of MCL-1 and BCL-2 in MM Cells

In an attempt to elucidate mechanisms triggered by the stromal BM microenvironment that could be modifying the activity of S63845 and venetoclax, we subsequently analyzed the expression of MCL-1 and BCL-2 anti-apoptotic proteins in MM.1S cells cultured for 48 h in direct contact with pMSCs isolated from four MM patients (Figure 1C). The co-culture with pMSCs induced a discrete but consistent increase in the expression of MCL-1 and a decrease in BCL-2 levels in MM cells relative to MM.1S cells in monoculture.

We also evaluated the expression of MCL-1 and BCL-2 proteins in a series of five MM cell lines co-cultured with pMSCs under the same conditions (Figure 1D). Similar to MM.1S cells, RPMI-8226 and NCI-H929 cell lines showed augmented MCL-1 protein levels when co-cultured with pMSCs. However, no noticeable changes in MCL-1 expression were observed in the JJN3 cell line and only a slight decrease was noticed in KMS12-BM cells. BCL-2 protein levels, contrary to MCL-1, were reduced in MM.1S, RPMI8226, KMS12-BM, and NCI-H929 cell lines in co-culture with pMSCs as compared with cells in monoculture. In JJN3 cells, BCL-2 expression remained unchanged.

### 3.3. The BM Stromal Microenvironment Deregulates the Expression of miRNAs Potentially Modulating MCL-1 or BCL-2 Levels in MM.1S Cells

To gain insight into possible mechanisms by which pMSCs could be modifying the expression of MCL-1 and BCL-2 proteins in MM cells and eventually affecting the efficacy of S63845 and venetoclax, we focused on post-transcriptional regulation of anti-apoptotic proteins by miRNAs. First, we studied changes produced in the miRNA expression profile of MM.1S when this cell line was co-cultured with pMSCs for 48 h (Affymetrix GeneChip miRNA 4.0 Array; unpublished data from our group). We then employed the Target Scan algorithm intending to identify potential miRNAs targeting MCL1 and BCL2 mRNAs. The bioinformatic analysis predicted a total of 53 miRNAs with an evolutionary conserved binding site in the 3’UTR of MCL1 mRNA and 58 in that of BCL2 (Table 1).

From the predicted miRNAs targeting MCL1 mRNA, we identified four (miR-193b-3p, miR-17-5p, miR-93-5p, and miR-106a-5p) with a significantly reduced expression in MM.1S cells after 48 h of co-culture with pMSCs in the miRNA microarray data set, with miR-193b-3p being the most significantly deregulated (Figure S2A). On the other hand, miR-21-5p was the only miRNA that by bioinformatic prediction targeted BCL2 mRNA and was found to be significantly upregulated in MM cells after their interaction with pMSCs (Figure S2B).

Subsequently, the significantly lower miR-193b-3p (Figure 1E) and higher miR-21-5p (Figure 1F) expressions detected in MM.1S cells in co-culture with pMSCs as compared with MM.1S cells in monoculture by microarrays were confirmed by qRT-PCR analysis. The expression of miR-193b-3p and miR-21-5p was also assessed in other MM cell lines, both in monoculture and co-culture conditions. RPMI8226 cells showed similar results to those observed in the MM.1S cell line. However, KMS12-BM and JJN3 cells only exhibited an increase in miR-21-5p after co-culture with pMSCs (Figure S3A,B). 

### 3.4. Changes in MCL-1 and BCL-2 Protein Expression Induced by pMSCs in MM Cells Are Partially Mediated by miR-193 and miR-21

**Figure 2:**
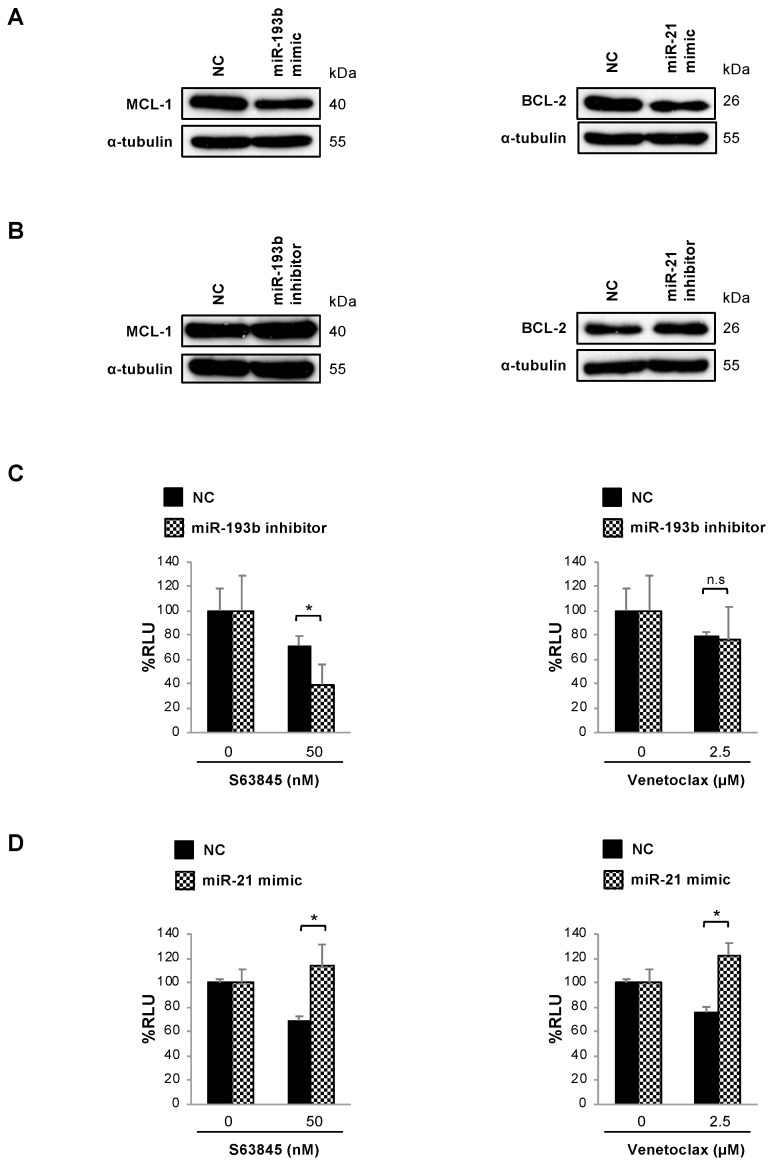
miR-193b-3p and miR-21-5p modulate MCL-1 and BCL-2 protein expression and S63845 and venetoclax activity in MM.1S cells. (**A**,**B**) Western blot evaluation of MCL-1 and BCL-2 in MM.1S cells respectively transfected with miR-193b-3p and miR-21-5p or non-targeting control (NC) mimics (**A**) or inhibitors (**B**). (**C**) MM.1S-luc cells were transiently transfected with miR-193b-3p inhibitor or NC and treated with S63845 50 nM or venetoclax 2.5 µM for 48 h. MM cell viability was assessed by bioluminescence, which was normalized relative to the growth of NC-transfected untreated cells. Results are expressed as the mean ± SD (*n* = 3). Student’s *t*-test (*, *p* < 0.05). (**D**) MM.1S-luc cells were transiently transfected with miR-21-5p mimic or NC and treated with S63845 50 nM or venetoclax 2.5 µM for 48 h. Cell viability was evaluated as in (**C**).

To study the putative role of miR-193b-3p and miR-21-5p as regulators of MCL-1 and BCL-2 expression, the MM.1S cell line was transiently transfected with correspondent miRNA mimics or inhibitors, and the expression of anti-apoptotic proteins was determined 48 h post-transfection. Importantly, MM.1S cells transfected with miR-193b-3p or miR-21-5p mimics (Figure 2A) showed decreased MCL-1 and BCL-2 levels, as compared with negative control (NC)-transfected cells. By contrast, MCL-1 and BCL-2 expressions were respectively increased upon transfection with miR-193b-3p and miR-21-5p inhibitors (Figure 2B). Taken together, these results seem indicative of miR-193b-3p and miR-21-5p negatively modulating the expression of MCL-1 and BCL-2.

To establish a functional link between the diminished expression of miR-193b-3p or the augmented expression of miR-21-5p in MM cells in co-culture with pMSCs and the altered S63845 and venetoclax cytotoxic effect observed in these conditions, MM.1S-luc cells were transiently transfected with miR-193b-3p inhibitors or miR-21-5p mimics and their respective NCs. Subsequently, cells were treated with S63845 50 nM or venetoclax 2.5 µM, and bioluminescence was measured 48 h post transfection. In concordance with results obtained in the presence of the stroma, mir-193b-3p inhibitor significantly increased S63845 efficacy as compared with NC, whereas no significant changes in venetoclax activity were observed (Figure 2C). In the same line, after the overexpression of miR-21-5p, a general decrease in both venetoclax and S63845 efficacies was observed with respect to NCs (Figure 2D).

### 3.5. MCL1 mRNA Is Directly Regulated by miR-193b, Whereas BCL2 Transcript Is Not Targeted by miR-21

**Figure 3:**
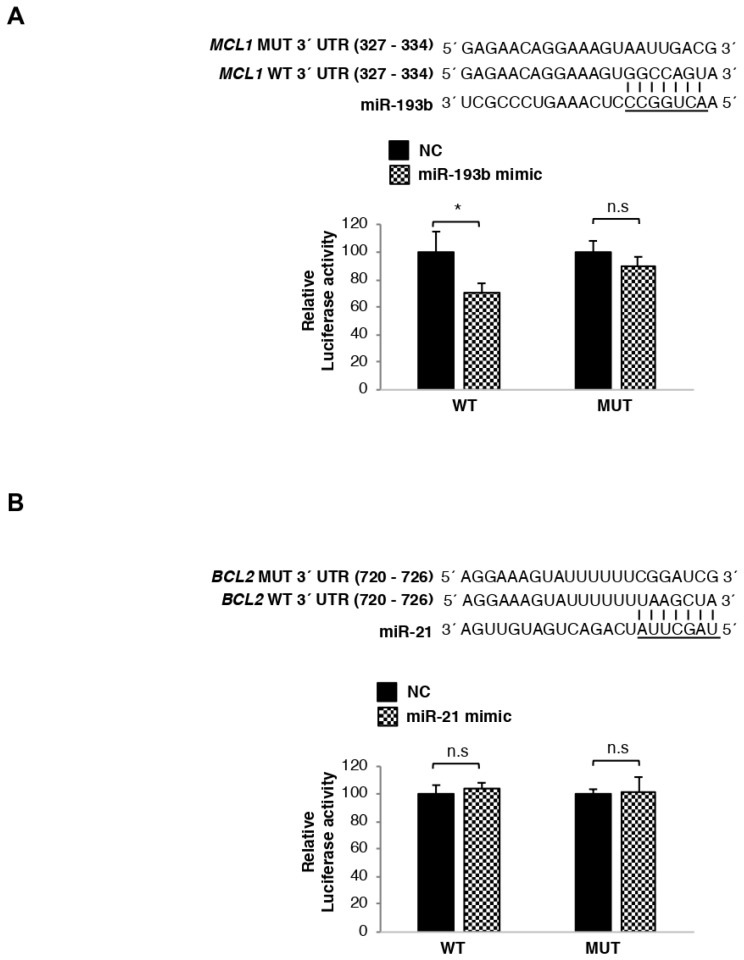
miR-193b-3p directly binds MCL1 mRNA inducing its degradation, whereas mir-21-5p is not directly regulating BCL2 mRNA expression. (**A**,**B**) Luciferase activity measured in HEK293 cells co-transfected with miR-193b-3p (**A**) miR-21-5p (**B**) or NC mimics and pmiR-Glo plasmids containing the wild-type (WT) or the mutant (MUT) miRNAs binding site of the 3′UTR MCL1 (**A**) BCL2 (**B**) genes cloned downstream of the luciferase reporter gene. Luciferase activity was normalized using renilla. All results are presented as the means ± SD of three different experiments. Significant differences with respect to cells transfected with NC were assessed with the Student’s *t*-test (*, *p* < 0.05).

To validate the MCL1 transcript as a target of miR-193b-3p and BCL2 mRNA as a target of miR-21-5p, luciferase reporter assays were performed using miRNA binding sites in their 3′UTR region. MCL1 and BCL2 wild-type (WT) 3′UTR sequences, respectively containing miR-193b-3p and miR-21-5p binding sites, were cloned into a dual-luciferase reporter plasmid. In parallel, 3′UTR sequences harboring mutant (MUT) binding sites were cloned into the same reporter plasmid and used as negative controls. HEK293 cells were co-transfected with WT or MUT constructs and the corresponding miRNA mimic or negative control (NC). The expression of luciferase activity was measured 24 h post transfection. Luciferase activity of cells co-transfected with MCL1 WT 3′UTR and miR-193b-3p mimics was significantly lower (*p* < 0.05) than that exhibited by MM.1S cells transfected with the NC miRNAs. By contrast, luciferase activity of transfected MUT constructs was not significantly affected by the presence of miR-193b-3p (Figure 3A). On the other hand, no reduction in luciferase activity was observed in cells co-transfected with either BCL2 WT or MUT 3′UTR constructs and miR-21-5p mimics (Figure 3B). Taken together, these data indicate that miR-193b-3p binds to the 3′UTR of MCL1 precluding its translation into protein, and thus the MCL1 transcript is a direct target of miR-193b-3p. However, miR-21-5p does not bind to the 3′UTR of BCL2, indicating that it is not directly modulating BCL-2 protein expression.

### 3.6. The Presence of Stromal Cells Modifies Interactions of MCL-1 and BCL-2 with BIM in Untreated and S63845- or Venetoclax-Treated MM.1S Cells 

**Figure 4:**
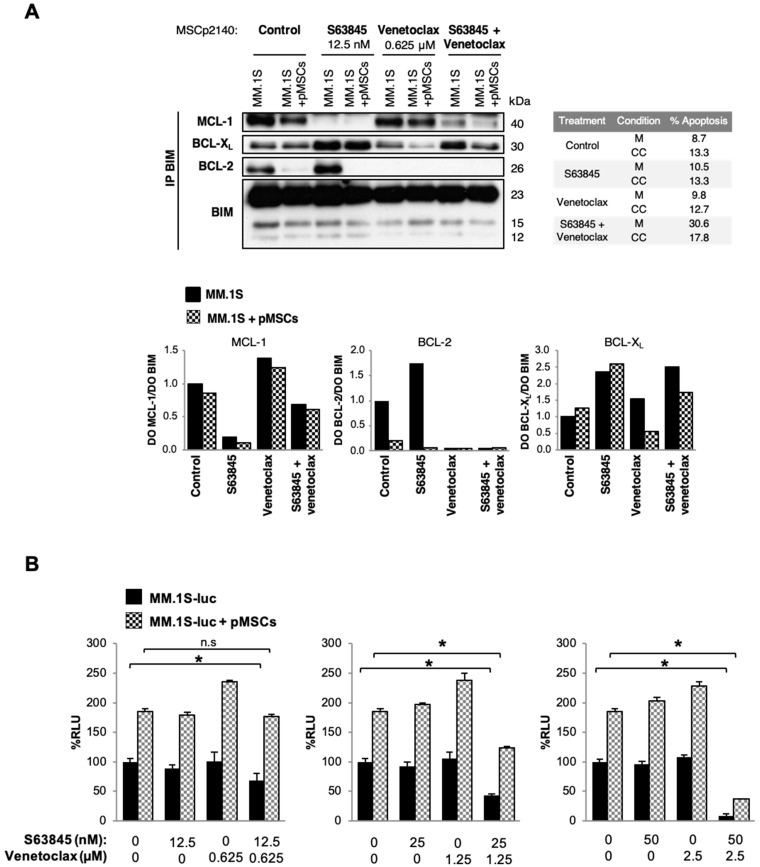
The stromal microenvironment modifies the mechanism of action and the efficacy of S63845 and venetoclax alone and in combination. (**A**) MM.1S cells were cultured in the absence or presence of pMSCs treated with S63845 and venetoclax alone or in combination for 48 h, and protein lysates were subjected to immunoprecipitation with an anti-BIM antibody. MCL-1, BCL-2, and BCL-X_L_ bound to BIM were then analyzed by immunoblotting. Their levels were quantified by densitometry analysis of bands (using ImageJ software), normalized to those of BIM, and depicted as bar diagrams. Percentages of apoptosis obtained after each of the treatments are shown in the adjacent table. (**B**) MM.1S-luc cells were co-cultured with pMSCs for 48 h with the double combination at the indicated doses. MM.1S-luc growth was assessed by the luciferase bioluminescence signal, which was normalized relative to the growth of MM.1S-luc cells alone and in the absence of drug treatment. Graphs show the mean ± SD (*n* = 3). Significant differences between the combination and untreated cells were assessed with the Student’s *t*-test (*, *p* < 0.05). M = monoculture; CC = co-culture.

Considering changes induced by pMSCs in the expression of MCL-1 and BCL-2 proteins and their influence on S63845 and venetoclax efficacy, we next investigated whether pMSCs were also affecting interactions of these anti-apoptotic proteins with the pro-apoptotic protein BIM. For that purpose, MM.1S cells were cultured alone or in the presence of pMSCs and exposed or not to S63845 and venetoclax. After 48 h, apoptosis induction was evaluated by flow cytometry and immunoprecipitation assays were performed (Figure 4A). 

In untreated cells, despite the increased MCL-1 expression previously observed in total lysates, MCL-1/BIM complexes were slightly diminished in the presence of pMSCs. On the other hand, the decreased BCL-2 total protein levels observed in MM.1S cells when co-cultured with pMSCs were accompanied by a drop in the levels of BCL-2 bound to BIM. Given that decreased interactions of BCL-2 with BIM in co-culture conditions did not result in the formation of extra MCL-1/BIM complexes, we sought to investigate the anti-apoptotic protein BCL-X_L_, non-targeted by any of the BH3-mimetics assessed in this study. In fact, the reduced amount of BCL-2/BIM complexes detected in the presence of pMSCs seemed to be accompanied by the formation of some additional BCL-X_L_/BIM complexes.

To explore stromal-mediated mechanisms of resistance based on interactions between anti-apoptotic proteins and BIM in treated cells, we employed concentrations of S63845 and venetoclax that did not substantially affect MM cell viability. Under treatment with S63845, interactions of MCL-1 with BIM were impaired in MM.1S cells both in monoculture and in co-culture with pMSCs. However, no increase in BCL-2/BIM complexes was detected in MM cells cultured in the presence of the stroma and exposed to S63845 as compared with non-treated cells. Of note, a noticeable increase in BCL-X_L_/BIM complexes was observed with S63845 in mono- and co-culture conditions. On the other hand, with venetoclax treatment, the interaction between BCL-2 and BIM was completely impaired, leading to an increase in MCL-1/BIM complexes in MM cells, either in monoculture or co-culture with pMSCs, as compared with untreated cells. However, BCL-X_L_/BIM levels were similar (monoculture) or even decreased (co-culture) compared with untreated cells. 

Finally, our group has previously demonstrated that the simultaneous treatment of MM cells with S63845 and venetoclax is a promising strategy for improved efficacy and for overcoming resistance to each of these agents in monotherapy [26]. Consequently, we also wanted to investigate the effect of the stromal BM microenvironment on S63845 in combination with venetoclax. The S63845 + venetoclax combination completely blocked interactions of BCL-2 with BIM and precluded the formation of additional compensatory MCL-1/BIM complexes, which were induced by venetoclax in monotherapy. However, interactions of BCL-X_L_ with BIM were also augmented with the combinatorial approach, although not to a further extent than with drugs in monotherapy.

### 3.7. S63845 Potently Synergizes with Venetoclax in the Presence of pMSCs

Because of our results, we subsequently analyzed whether the S63845 + venetoclax combination was synergistic in MM.1S cells in co-culture with pMSCs. Therefore, MM.1S-luc cells co-cultured with pMSCs were treated with increasing concentrations of S63845 and venetoclax alone and in combination for 48 h, and tumor cell viability was measured by bioluminescence (Figure 4B). The double combination had a strong anti-myeloma effect even in the presence of the tumor-associated stromal microenvironment, demonstrating its superiority over S63845 and venetoclax in monotherapy and co-culture with pMSCs. Similarly, the protective effect of pMSCs was evaluated in other MM cell lines (RPMI8226, KMS12-BM, and JJN3) treated with this combination (Figure S4). Except for JJN3, the stroma was observed to confer apoptosis resistance at lower doses. However, this stroma-mediated resistance was overcome when combining concentrations of both drugs only able to modestly kill MM cells in monotherapy and co-culture conditions. As was observed with the drugs in monotherapy, the double combination did not affect pMSCs’ viability (Figure S5).

## 4. Discussion

Due to the deregulated expression of the BCL-2 protein family in B-cell malignancies (with increased expression of anti-apoptotic proteins and downregulation of pro-apoptotic members) [30], BH3-mimetics are emerging as promising therapeutic options for these diseases. This relies on B cell dyscrasias being relatively “primed for apoptosis” as compared with their normal counterparts, with anti-apoptotic proteins being engaged in sequestering high levels of pro-apoptotic proteins to ensure survival [15,31,32]. Under these circumstances, BH3-mimetics are capable of binding with high affinity to specific anti-apoptotic proteins releasing pro-apoptotic members to tip the balance towards apoptosis. In some hematological malignancies, such as chronic lymphocytic leukemia, cells mostly express the BH3-only proteins BIM and PUMA, which are constitutively bound to BCL-2. This makes CLL cells highly sensitive to BCL-2-selective BH3-mimetics such as venetoclax [15]. In MM, however, BH3 profiling [33,34] has revealed tumor-cell dependency on anti-apoptotic proteins to be highly heterogeneous, with primary MM cells at diagnosis being dependent on MCL-1 or BCL-2, or co-dependent on either BCL-2/MCL-1 or BCL-X_L_/MCL-1 [34]. In particular, the MM.1S cell line has been reported to have an intermediate dependence on MCL-1, high dependence on BCL-X_L_, and low dependence on BCL-2 [34]. Regarding the influence of the BM microenvironment on the BCL-2 family of proteins, IL6-mediated signals have been shown to promote MCL-1 overexpression and dependence in MM [27,35], with phosphorylation of BIM shifting its binding from BCL-2 and BCL-X_L_ to MCL-1 [17].

In our hands, the direct co-culture with primary pMSCs increased total MCL-1 protein levels in MM.1S, RPMI8226, and NCI-H929, but not in KMS12-BM and JJN3 cells. By contrast, the presence of stromal cells induced a decrease in BCL-2 expression in all cell lines except for JJN3. Interestingly, the increased expression of MCL-1 was found to be associated with concomitantly reduced levels of miR-193b-3p. We demonstrate that the inhibition of miR-193b-3p in MM.1S cells in monoculture, conveying the co-culture condition, induced the overexpression of MCL-1. Consistently, the MCL1 transcript was later corroborated as a direct target of miR-193b-3p. In concordance with these results, miR-193a belonging to the same miRNA family as miR-193b has also been shown to directly target MCL1 mRNA in the context of dexamethasone-resistant MM cell lines [36] and colorectal cancer [37]. Furthermore, we observed that transfection of the miR-193b inhibitor in MM cells in monoculture increased S63845 efficacy. In this regard, a higher sensitivity to S63845 has been previously associated with MCL-1 overexpression in MM cells harboring 1q amplifications, where the MCL1 locus resides [26,38]. No differences in the activity of venetoclax were detected upon miR-193b inhibition.

In relation to BCL-2 expression, we found that its reduced levels after interaction with pMSCs was associated with increased miR-21-5p expression in almost all MM cell lines tested. However, miR-21-5p was not found to directly bind to the BCL2 3′UTR mRNA in luciferase reporter assays. Contradictory findings of whether mir-21 positively or negatively regulates BCL-2 expression have been published in other tumors [39,40,41,42,43]. In MM, our results only suggest an indirect negative regulation of BCL-2 upon miR-21-5p overexpression. In addition, the general decrease in S63845 and venetoclax activities observed after miR-21-5p upregulation in MM cells in monoculture suggests an involvement of this miRNA in a more general stroma-mediated mechanism of resistance.

Besides the expression of anti-apoptotic proteins, the co-culture with pMSCs also modified their interactions with the pro-apoptotic BIM protein in untreated MM cells. Despite the augmented MCL-1 total protein levels observed in MM.1S cells in direct co-culture with primary pMSCs, an increase in MCL-1/BIM complexes was not detected. Similar results indicating that the overexpression of MCL-1 in some MM cell lines, including MM.1S, does not result in augmented MCL-1/BIM complexes have been reported by other groups [44]. It is noticeable that the reduced BCL-2 expression observed in MM.1S cells after direct co-culture with primary pMSCs was accompanied by a decrease in the interactions of BCL-2 with BIM. In contrast, an increase in BCL-X_L_/BIM complexes was observed. These results suggest that in untreated MM.1S cells, the direct contact with pMSCs keeps MCL-1 cell dependence unchanged while inducing a shift from BCL-2 to BCL-X_L_ dependence.

Given the later data, we hypothesized that the presence of the BM stroma would alter interactions between anti-apoptotic proteins and BIM in MM cells treated with S63845 and venetoclax. For this purpose, doses of both drugs not exerting substantial apoptosis alone or in combination were used. Importantly, S63845 and venetoclax remained active in the presence of pMSCs, being able to impair the interactions of their respective targets with the pro-apoptotic BIM protein. We also observed that consistent with the shift from BCL-2 to BCL-X_L_ dependence observed in untreated MM.1S cells when co-cultured in the presence of pMSCs, the mechanism of resistance to S63845 treatment in the presence of the BM microenvironment seemed to be mediated by the formation of additional BCL-X_L_/BIM, but not BCL-2/BIM complexes. However, resistance to venetoclax was mainly mediated by increased MCL-1/BIM levels in co-culture conditions. Finally, the S63845 + venetoclax combination was also assessed, and although MCL-1/BIM and BCL-2/BIM complexes were diminished as compared with untreated cells, an increase in the binding of BCL-X_L_ to BIM was observed, thus potentially shifting dependency towards BCL-X_L_.

Moreover, we showed that despite the shift of dependency towards BCL-X_L_ observed when MCL-1 and BCL-2 were simultaneously inhibited, the S63845 + venetoclax combination was highly effective regardless of MM.1S cells being in monoculture or co-culture with pMSCs. This is in line with previous observations reported by our group and others, showing high synergism for the mentioned combination in vitro, ex vivo, and in vivo [38,39]. Whether the use of combinations of BH3-mimetics targeting MCL-1 and BCL-2 may be appropriate for MM patients probably depends on the adequate management of hematologic and cardiac toxicities [15].

In conclusion, we have shown that co-culture with pMSCs generally alters the expression of anti-apoptotic proteins BCL-2 and MCL-1 in several MM cell lines. The upregulated expression of MCL-1 observed in MM.1S cells cultured in the presence of the stroma is, at least partially mediated through the reduced expression of miR-193b-3p induced in these conditions. Decreased BCL-2 levels in MM.1S cells in co-culture with pMSCs were accompanied by increased miR-21-5p expression, although BCL2 transcripts were not confirmed as a direct target of this miRNA. Mechanistically, we have shown that BCL-X_L_/BIM complexes may play a role in the development of resistance to S63845 in MM.1S in co-culture with pMSCs. On the other hand, the resistance to venetoclax in the presence of the stroma seemed to be mainly mediated by increased interactions of MCL-1 with BIM. Finally, the S63845 + venetoclax combination in the co-culture setting was highly effective and overcamethe co-dependencies for anti-apoptotic proteins observed in these conditions.

## Figures and Tables

**Table 1:** MicroRNAs with evolutionary conserved binding sites in the 3′ UTR of the indicated mRNAs among mammals.

MCL1 mRNA	BCL2 mRNA
hsa-miR-125a-5p	hsa-miR-16-5p
hsa-miR-4319	hsa-miR-6838-5p
hsa-miR-125b-5p	hsa-miR-195-5p
hsa-miR-29a-3p	hsa-miR-424-5p
hsa-miR-29b-3p	hsa-miR-15a-5p
hsa-miR-29c-3p	hsa-miR-15b-5p
hsa-miR-526b-3p	hsa-miR-497-5p
hsa-miR-106a-5p	hsa-miR-4262
hsa-miR-20b-5p	hsa-miR-181d-5p
hsa-miR-93-5p	hsa-miR-181b-5p
hsa-miR-519d-3p	hsa-miR-181a-5p
hsa-miR-17-5p	hsa-miR-181c-5p
hsa-miR-20a-5p	hsa-miR-125a-5p
hsa-miR-106b-5p	hsa-miR-125b-5p
hsa-miR-520f-3p	hsa-miR-4319
hsa-miR-302c-3p.2	hsa-miR-153-3p
hsa-miR-133a-3p.1	hsa-miR-182-5p
hsa-miR-133b	hsa-miR-30d-5p
hsa-miR-133a-3p.2	hsa-miR-30e-5p
hsa-miR-373-3p	hsa-miR-30a-5p
hsa-miR-372-3p	hsa-miR-30b-5p
hsa-miR-520a-3p	hsa-miR-30c-5p
hsa-miR-520d-3p	hsa-miR-449a
hsa-miR-520e	hsa-miR-449b-5p
hsa-miR-302d-3p	hsa-miR-34a-5p
hsa-miR-302b-3p	hsa-miR-34c-5p
hsa-miR-302c-3p.1	hsa-miR-96-5p
hsa-miR-520c-3p	hsa-miR-1271-5p
hsa-miR-302a-3p	hsa-miR-200b-3p
hsa-miR-520b	hsa-miR-429
hsa-miR-302e	hsa-miR-200c-3p
hsa-miR-135a-5p	hsa-miR-365b-3p
hsa-miR-135b-5p	hsa-miR-365a-3p
hsa-miR-153-3p	hsa-miR-204-5p
hsa-miR-4262	hsa-miR-211-5p
hsa-miR-181b-5p	hsa-miR-140-3p.2
hsa-miR-181a-5p	hsa-miR-21-5p
hsa-miR-181d-5p	hsa-miR-590-5p
hsa-miR-181c-5p	hsa-miR-6088
hsa-miR-5590-3p	hsa-miR-143-3p
hsa-miR-142-5p	hsa-miR-4770
hsa-miR-193a-3p	hsa-miR-383-5p.2
hsa-miR-193b-3p	hsa-miR-383-5p.1
hsa-miR-4465	hsa-miR-23b-3p
hsa-miR-26b-5p	hsa-miR-130a-5p
hsa-miR-26a-5p	hsa-miR-23c
hsa-miR-1297	hsa-miR-23a-3p
hsa-miR-101-3p.1	hsa-miR-202-5p
hsa-miR-101-3p.2	hsa-miR-448
hsa-miR-325-3p	hsa-miR-342-3p
hsa-miR-873-5p.1	hsa-miR-503-5p
hsa-miR-381-3p	hsa-miR-382-3p
hsa-miR-300	hsa-miR-219a-2-3p
	hsa-miR-374c-5p
	hsa-miR-655-3p
	hsa-miR-323a-3p
	hsa-miR-6835-3p
	hsa-miR-224-5p

## Data Availability

The data presented in this study are available in the article or in the Supplementary material.

## Supplementary Materials

The following are available online at https://www.mdpi.com/2073-4409/10/3/559/s1, Figure S1: S63845 and venetoclax do not reduce pMSC’s viability, Figure S2: Normalized expression signal of the miRNAs that are significantly deregulated in MM.1S cells after co-culture with pMSCs (MM1S + MSCp) versus MM.1S cells in monoculture, Figure S3: pMSCs modify the expression of miR-193b-3p and miR-21-5p in different MM cell lines, Figure S4: The stromal microenvironment modifies the efficacy of S63845 in combination with venetoclax in different MM cell lines, Figure S5: S63845 in combination with venetoclax does not reduce pMSCs’ viability.
