# Supplementary material for: Stroma-Mediated Resistance to S63845 and Venetoclax through MCL-1 and BCL-2 Expression Changes Induced by miR-193b-3p and miR-21-5p Dysregulation in Multiple Myeloma
Source: Cells. 2021 Mar 4;10(3):559. doi: 10.3390/cells10030559 (PMC8001939; doi:10.3390/cells10030559)
Supplement: Supplementary file 1 [file cells-10-00559-s001.pdf]

SUPPLEMENTARY FIGURE 1

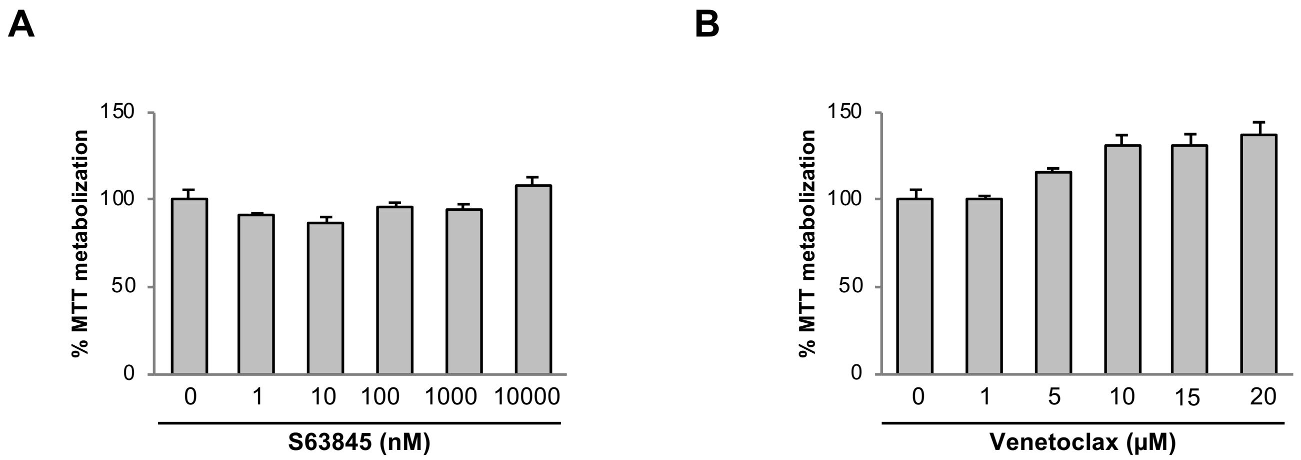

Supplementary Figure 1. S63845 and venetoclax do not reduce pMSC viability. (A-B) pMSCs isolated from a MM patient were treated with increasing doses of S63845 (A) or venetoclax (B) for 48 hours, and cell viability was measured by the MTT assay. Average absorbances relative to the percentage of the control are shown. Data presented are means ± SD (n=3).

SUPPLEMENTARY FIGURE 2

A

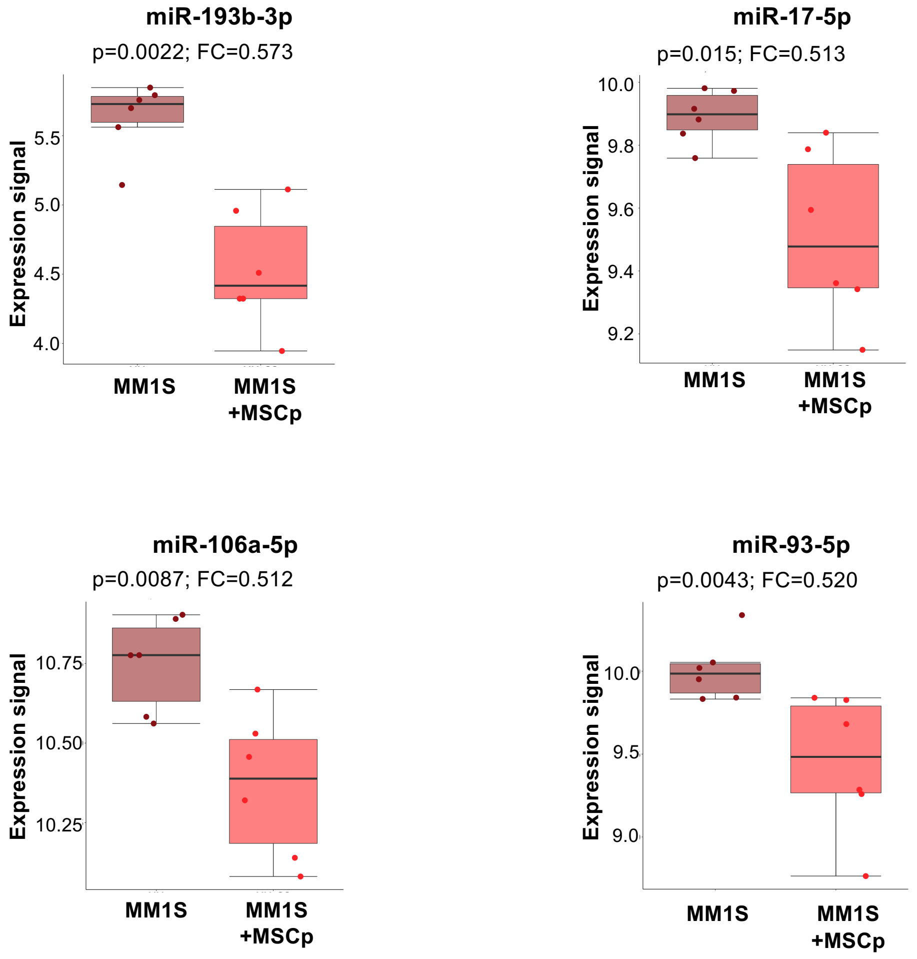

B

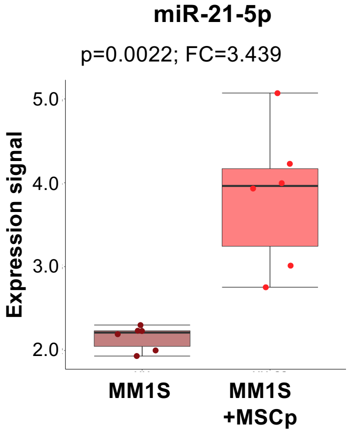

Supplementary Figure 2. Normalized expression signal of the miRNAs that are significatively deregulated in MM.1S cells after co-culture with pMSCs (MM1S+MSCp) *versus* MM.1S cells in monoculture. The expression of miRNAs was measured with *Affymetrix* GeneChip miRNA 4.0 Array (unpublished data); boxplots represent selected miRNAs which were predicted to target MCL1 (A) and BCL2 (B) transcripts by *TargetScan* algorithm.

SUPPLEMENTARY FIGURE 3

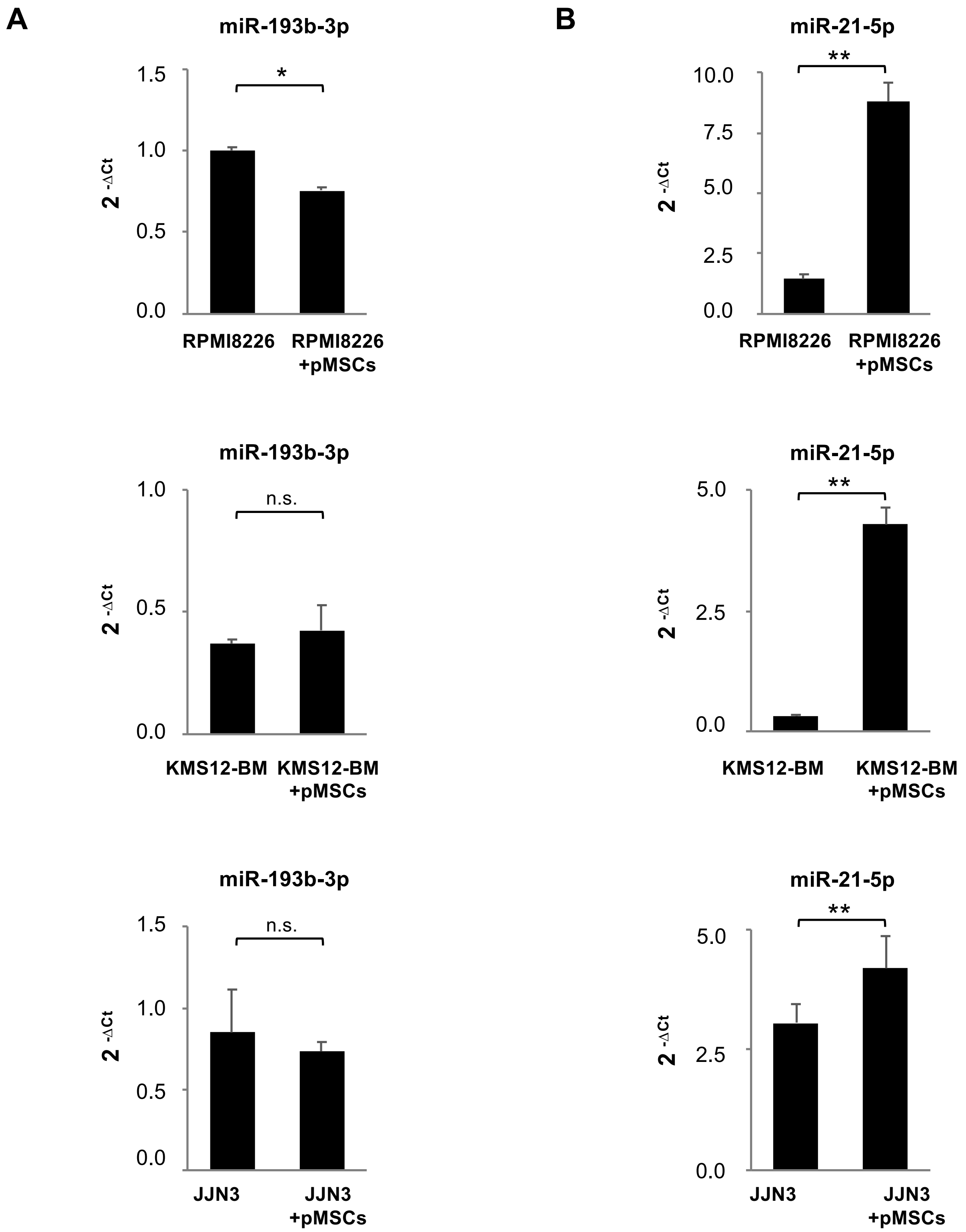

Supplementary Figure 3. pMSCs modify the expression of miR-193b-3p and miR-21-5p on different MM cell lines. Normalized expression of miR-193b-3p (A) and miR-21-5p (B) in RPMI8226, KMS12-BM and JJN3 cells alone or co-cultured with pMSCs as assessed by qRT-PCR. Results are expressed as the mean ± SEM. Significant differences were assessed with the Student t test (*, $p < 0.05$; **, $p < 0.01$)

SUPPLEMENTARY FIGURE 4

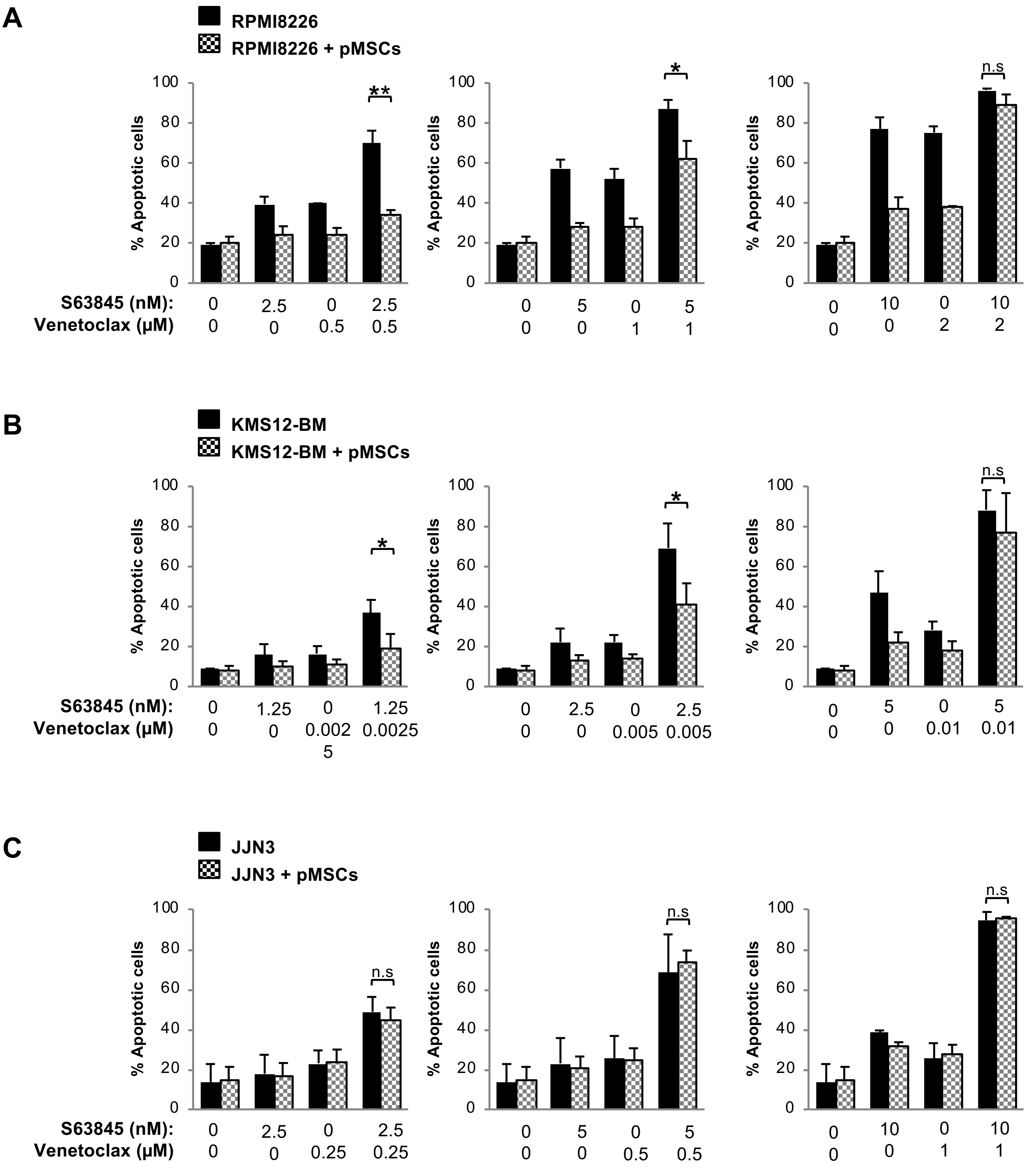

Supplementary Figure 4. The stromal microenvironment modifies the efficacy of S63845 in combination with venetoclax in different MM cell lines. RPMI8226 (A), KMS12-BM (B) and JJN3 (C) cells were co-cultured with pMSCs with the double combination at the indicated doses. After, 48 hours, apoptosis induction on MM cells was analyzed by flow cytometry after Annexin-V and PI staining. Data represent the percentage of apoptotic cells for each condition ± SD. Significant differences between cells in monoculture and in co-culture treated with the combination were assessed with the Student t test (*, $p < 0.05$; **, $p < 0.01$).

SUPPLEMENTARY FIGURE 5

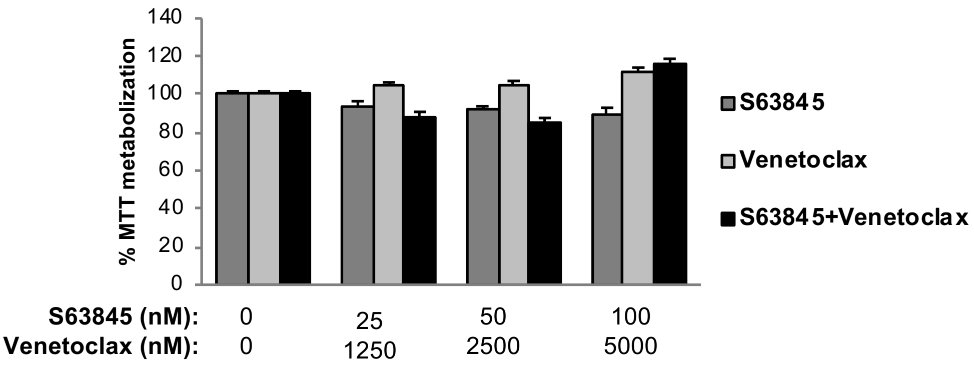

Supplementary Figure 5. S63845 in combination with venetoclax does not reduce pMSCs viability. pMSCs isolated from a MM patient were treated with increasing doses of the S63845 + venetoclax combination for 48 hours, and cell viability was measured by MTT assay. Average absorbances relative to the percentage of the untreated control are shown. Data presented are means ± SD (n=3).
